# Development of a reverse transcription-loop-mediated isothermal amplification as a rapid early-detection method for novel SARS-CoV-2

**DOI:** 10.1080/22221751.2020.1756698

**Published:** 2020-05-18

**Authors:** Yun Hee Baek, Jihye Um, Khristine Joy C. Antigua, Ji-Hyun Park, Yeonjae Kim, Sol Oh, Young-il Kim, Won-Suk Choi, Seong Gyu Kim, Ju Hwan Jeong, Bum Sik Chin, Halcyon Dawn G. Nicolas, Ji-Young Ahn, Kyeong Seob Shin, Young Ki Choi, Jun-Sun Park, Min-Suk Song

**Affiliations:** a Department of Microbiology, Chungbuk National University College of Medicine and Medical Research Institute, Cheongju, Republic of Korea; b Research Institute of Public Health, National Medical Center, Seoul, Republic of Korea; c Center for Infectious Diseases Research, Department of Internal Medicine, National Medical Center, Seoul, Republic of Korea; d School of Biological Sciences, Chungbuk National University, Cheongju, Republic of Korea; e Department of Laboratory Medicine, Chungbuk National University College of Medicine, Cheongju, Republic of Korea

**Keywords:** SARS-CoV-2, COVID-19, reverse transcription-loop-mediated isothermal amplification, molecular diagnosis, colorimetric detection, novel coronavirus

## Abstract

The previous outbreaks of SARS-CoV and MERS-CoV have led researchers to study the role of diagnostics in impediment of further spread and transmission. With the recent emergence of the novel SARS-CoV-2, the availability of rapid, sensitive, and reliable diagnostic methods is essential for disease control. Hence, we have developed a reverse transcription loop-mediated isothermal amplification (RT-LAMP) assay for the specific detection of SARS-CoV-2. The primer sets for RT-LAMP assay were designed to target the nucleocapsid gene of the viral RNA, and displayed a detection limit of 10^2^ RNA copies close to that of qRT-PCR. Notably, the assay has exhibited a rapid detection span of 30 min combined with the colorimetric visualization. This test can detect specifically viral RNAs of the SARS-CoV-2 with no cross-reactivity to related coronaviruses, such as HCoV-229E, HCoV-NL63, HCoV-OC43, and MERS-CoV as well as human infectious influenza viruses (type B, H1N1pdm, H3N2, H5N1, H5N6, H5N8, and H7N9), and other respiratory disease-causing viruses (RSVA, RSVB, ADV, PIV, MPV, and HRV). Furthermore, the developed RT-LAMP assay has been evaluated using specimens collected from COVID-19 patients that exhibited high agreement to the qRT-PCR. Our RT-LAMP assay is simple to perform, less expensive, time-efficient, and can be used in clinical laboratories for preliminary detection of SARS-CoV-2 in suspected patients. In addition to the high sensitivity and specificity, this isothermal amplification conjugated with a single-tube colorimetric detection method may contribute to the public health responses and disease control, especially in the areas with limited laboratory capacities.

## Introduction

Among the coronaviruses, the emergence of severe acute respiratory syndrome coronavirus (SARS-CoV) and Middle East respiratory syndrome coronavirus (MERS-CoV) have caused threats to public health in 2002 and 2012 [1–10]. By the end of December 2019, another zoonotic human coronavirus has emerged in Wuhan, Hubei Province of China [5,10–20]. Initially, 27 patients with clinical manifestations of pneumonia, fever, difficulty in breathing, and chest radiographs with bilateral lung infiltrative lesions were observed [10–13,16,20–22]. All patients were clinically tested negative for both the MERS-CoV and SARS-CoV [13]. An abrupt increase in cases of viral pneumonia of unknown etiology led the Chinese authorities to report the situation to the World Health Organization (WHO) on December 31, 2019 [11,12]. During the ongoing scientific investigations, WHO initially proposed the interim name of this novel virus as “2019-nCoV,” but later, COVID-19 was designated as the official name of this disease [23]. Following a thorough assessment of phylogeny, taxonomy, and established practice, the International Committee on Taxonomy of Viruses (ICTV) formally recognized this virus to be related to SARS-CoVs, and designated this virus as “SARS-CoV-2” [24].

SARS-CoV-2 falls into the genus *betacoronavirus*, with a sequence homology of 80–89% with coronaviruses discovered in humans, bats, and other wild animals (for instance, SARS-CoV and bat SARS-like CoV) [3,5,7,16–18,20,22]. Due to an increase in the risk of a potential spread of this virus to countries with weaker health systems, the WHO have declared the SARS-CoV-2 outbreak as a disease of “public health emergency of international concern” [25]. Owing to the current disease situation, this novel coronavirus may remarkably become the third coronavirus posing significant threats to public health worldwide.

The impediment of further spread and transmission of this disease requires rapid and reliable identification, especially in areas with limited laboratory capacity. Concurrently, in hospitals, the identification of the causative agents of recurrent acute respiratory infections requires routine and confirmatory diagnosis through quantitative real-time polymerase chain reaction (qRT-PCR). In recent years, the loop-mediated isothermal amplification (LAMP) method that includes an exponential amplification of specific nucleic acid sequences at a constant temperature, has been widely utilized for the rapid detection of virus-specific genes [26,27]. The specificity and sensitivity of this method is generally comparable to those of the conventional PCR and qRT-PCR. The LAMP assays merged with reverse transcription steps have been developed for the detection of RNA viruses, including SARS-CoV, MERS-CoV, influenza, and other respiratory viruses [26–32].

In the present study, a single-tube reverse transcription loop-mediated isothermal amplification (RT-LAMP) assay has been developed for the detection of the SARS-CoV-2 nucleocapsid (N) gene through colorimetric visualization. The study was aimed to develop a rapid, simple and sensitive molecular detection assay for the novel coronavirus that can differentiate it from other currently circulating human coronaviruses, including OC43, 229E, NL64, and MERS-CoV as well as other respiratory viruses. This method enables the detection of SARS-CoV-2 within 30 min, excluding RNA extraction, with the detection limit of 10^2^ RNA copies which is close to qRT-PCR. Furthermore, by combining the amplification process with colorimetric detection, the assay is suitable for rapid and simple diagnosis within poorly equipped primary hospitals and laboratories, and in situations where an urgent diagnosis is needed.

## Materials and methods

### RT-LAMP primer designing

The RT-LAMP primers were designed for the detection of SARS-CoV-2 based upon the already available sequences in Global Initiative on Sharing All Influenza Data (GISAID) (www.gisaid.org). The sequences were aligned using CLC Genomics Workbench 10.0.1 (Qiagen, USA). A conserved region was identified in the target N gene sequence (*GenBank Accession no.: NC_045512,* position 28,285–28,529) that was used as a template for the designing of the RT-LAMP primers.

To identify the most efficient primer set, seven sets of specific RT-LAMP primers were initially designed using the PrimerExplorer V4 (Eiken Chemical Co. LTD, Tokyo, Japan) programme based upon the published sequence of the N gene. Primer sets included two external primers (forward outer primer F3 and backward outer primer B3), two internal primers (forward inner primer FIP and backward inner primer BIP), and two loop primers (backward loop primer LB and forward loop primer LF). All primers were synthesized by Bionics, Inc. (Republic of Korea). Table 1 represents the detailed information regarding all the designed primer sets. Further, 9 contributing laboratories are gratefully acknowledged for sharing their sequences and metadata in GISAID for designing primers for this assay (Supplementary Table 1).

**Table 1.** Primers for the detection of the novel Severe Acute Respiratory Syndrome-Coronavirus – 2 (SARS-CoV-2) through Reverse Transcription-Loop-Mediated Isothermal Amplification (RT-LAMP).

Target gene	Primer name	Sequence (5′-3′)	Primer final conc. (μM)	Gene position	Length (mer)
Nucleocapsid (N)	nCoV N-F3	TGGACCCCAAAATCAGCG	5	28285–28302	18
	nCoV N-B3	AGCCAATTTGGTCATCTGGA	5	28510–28529	20
	nCoV N-FIP	CGTTGTTTTGATCGCGCCCC-ATTACGTTTGGTGGACCCTC	20	28373–28392 + 28316–28335	40
	nCoV N-BIP	ATACTGCGTCTTGGTTCACCGC-ATTGGAACGCCTTGTCCTC	20	28416–28437 + 28476–28494	41
	nCoV N-LF	TGCGTTCTCCATTCTGGTTACT	5	28349–28370	22
	nCoV N-LB	TCTCACTCAACATGGCAAGGAA	5	28438–28459	22

### Viral RNA synthesis and extraction from cell-cultured viral samples

Initially, primers containing T7 RNA Polymerase promoter were designed using BetaCoV/Wuhan-Hu-1/2019 (GISAID, EPI_ISL_402125) sequence for SARS-CoV-2 and SARS-CoV/HKU-39849 (GenBank, Accession no.: AY278491.2) sequence, and outsourced from Bionics, Inc (Republic of Korea). The T7-flagged PCR product was then synthesized for *in vitro* transcription. The details of sequence and primers used for the RNA synthesis are provided in Supplementary Table 2. The artificial SARS-CoV-2 and SARS-CoV RNA was synthesized using MEGAscript® Kits (Invitrogen, USA) following the manufacturer’s instructions. RNA was recovered through lithium chloride precipitation and stored at −80 °C till further use. For quantification of RNA transcript, RNA copy number using EndMemo DNA/RNA Copy number calculator (http://endmemo.com/bio/dnacopynum.php) was calculated, quantitated using Qubit RNA HS Assay Kit (Thermo Fisher Scientific Inc., Massachusetts, USA) following the manufacturer’s instructions, and the end-point diluted RNA was confirmed through qRT-PCR.

Moreover, BetaCoV/Korea/NMC01/2020 and BetaCoV/Korea/NMC02/2020 viruses were propagated and passaged twice in Vero cells. Vero cells were maintained in Dulbecco’s modified Eagle’s medium (DMEM; Gibco-Invitrogen, Carlsbad, CA, USA) containing 10% fetal bovine serum (FBS) and 1% antibiotics, and incubated at 37 °C in 5% CO_2_. Intact viral RNA was extracted using RNeasy mini kit (Qiagen, USA) followed the manufacturer’s instructions. The viral RNA was stored at −80 °C till further use. The complete experimental work with SARS-CoV-2 was conducted in Enhanced Biosafety Level 3 (BL-3+) facility at Chungbuk National University as approved by the Korea Center for Disease Control.

### Optimization of the RT-LAMP reaction condition using in vitro transcribed RNA

To optimize the sensitivity and specificity of RT-LAMP detection, different primer concentrations (2.5–20 μM for external primer F3 and B3, 20–80 μM for internal primer FIP and BIP, and 5–40 μM for loop primer LF and LB) were tested. For a 10 μL RT-LAMP reaction, a master mix solution was prepared containing of WarmStart® Colorimetric LAMP 2X Master Mix (NEB, UK) (5 μL), F3 and B3 primers (1 μL each), FIP and BIP primers (1 μL each), and LF and LB primers (1 μL each). The optimized final concentrations of all primers are represented in Table 1.

The RNA template (2 μL) (extracted from virus and/or synthesized RNA) was added to the master mix and the RT-LAMP reaction was performed at 65 °C. Detection limit determination was performed by processing tenfold serially diluted (1 × 10^11^ to 1 × 10^0^) synthesized viral RNAs in the RT-LAMP. Concomitantly, same samples were subjected to qRT-PCR according to the shared protocol developed by the National Institute of Infectious Diseases of Japan, which showed one of the highest sensitivities in the comparison study [33,34]. Briefly, SARS-CoV-2 qRT-PCR was performed using iTaq Universal Probes One-Step Kit (Bio-Rad, Hercules, CA, USA) with conditions, including reverse transcription at 55 °C for 30 min, initial denaturation at 95 °C for 15 min, 40 cycles of denaturation at 95 °C for 15 s and annealing at 60 °C for 1 min using CFX96 TouchTM Real-Time PCR Detection System (Bio-Rad, Hercules, CA, USA). The cycle threshold (Ct) values in qRT-PCR analysis were determined based upon the set baseline threshold value of 100. The Ct values of in qRT-PCR results analysis were determined based upon the set baseline threshold value (100), validated by standard curve graph generated from serially diluted copies of templates with correlation coefficient value of 0.99.

To evaluate the time efficiency of the developed assay, optimization of the RT-LAMP reaction was done at varying reaction time points (10, 20, 30, 40, 50, and 60 min) using the determined limit concentration of viral RNA. Ten repeats of the RT-LAMP reaction were performed using the determined viral RNA concentrations with incubations of 30 and 60 min. For comparison, the detection limit of qRT-PCR was also determined by performing ten repeats of the reaction using 10^0^, 10^1^, 10^2^, and 10^3^ RNA copies. Positive RT-LAMP reactions resulted in a colour change of phenol red pH indicator from pink to yellow due to decreased pH in the presence of an extensive DNA polymerase activity. Thus, the results could be directly observed by naked eye. The results from RT-LAMP reactions were also confirmed through 2% agarose gel electrophoresis.

### Confirmation of the sensitivity and specificity of RT-LAMP for the detection of SARS-CoV-2 using intact viral RNA

The sensitivity of the developed RT-LAMP assay to detect SARS-CoV-2 was confirmed using the cell propagated cultures of SARS-CoV-2 that were grown and passaged twice in Vero cells. This intact viral RNA was ten-fold serially diluted to 10^−9^ and processed for parallel testing with RT-LAMP assay and qRT-PCR. The serially diluted RNA (2 μL) was mixed with the optimized master mix and subjected to RT-LAMP assay as described previously [32].

To determine the specificity of the optimized RT-LAMP assay for targeting SARS-CoV-2, the assay was tested across three-panel sets of different RNA samples that were extracted from patients’ nasal swabs and/or cell-propagated isolates of respiratory disease-causing agents. The RT-LAMP was cross-tested against a panel set of RNA samples of related coronaviruses, which includes human coronavirus 229E (229E), human coronavirus NL63 (NL63), human coronavirus OC43 (OC43), and Middle East Respiratory Syndrome coronavirus (MERS-CoV) tested using the developed RT-LAMP assay. The optimized RT-LAMP assay was also tested against a panel of human infecting and avian influenza viruses. The RNA samples of A/California/04/2009 (H1N1pdm), A/Perth/16/2009 (H3N2), B/Brisbane/60/2008 (Victoria lineage), and B/Phuket/3073/2013 (Yamagata lineage); highly pathogenic avian influenza viruses, including H5N1, H5N6, H5N8, and H7N9; and low pathogenic avian influenza viruses, including H2, H4∼H12 were also tested for any reactivity [32]. Furthermore, a panel set of other respiratory disease-causing viruses, including adenovirus (AdV), parainfluenza virus (PIV), human metapneumovirus (MPV), human bocavirus (HboV), human rhinovirus (HRV), respiratory syncytial virus A (RSVA), and respiratory syncytial virus B (RSVB) were tested. Clinical samples of human respiratory viruses were obtained from the Chungbuk National University Hospital in the Republic of Korea. The MERS-CoV Korean isolate (MERS-CoV/KOR/KNIH/002 _05_2015, GenBank, Accession no.: KT029139) was kindly provided by the Korea Centers for Disease Control and Prevention (KCDC). All work with MERS-CoV was conducted in Enhanced Biosafety Level 3 (BL-3+) facility approved by the Korea Center for Diseases Control at Chungbuk National University. The SARS-CoV-2 qRT-PCR was performed following the protocol used for the optimization and the sensitivity assay. All viruses used in the panel were also confirmed for viral RNA using one-step RT–PCR using their specific primers. The detailed information about the primers and PCR conditions used for these panel viruses is presented in Supplementary Table 3.

### Clinical evaluation of the RT-LAMP assay for SARS-CoV-2 detection

Fourteen nasal swabs collected from COVID-19 patients in the National Medical Center, Republic of Korea were processed for viral RNA extraction. Purified RNA was used for the clinical evaluation of the RT-LAMP assay in comparison to the qRT-PCR method as described above. An agreement between the two tests was also evaluated using Cohen’s Kappa.

### Ethics statement

Clinical samples were collected from the National Medical Center, South Korea. Samples were collected under the approved guidelines and relevant regulations. The Ethics Committee and Institutional Review Board of National Medical Center (IRB no. H-2002-111-002) approved all experimental procedures. All experiments were performed following the approved guidelines.

## Results

### RT-LAMP primer designing and standardization

**Figure 1.**
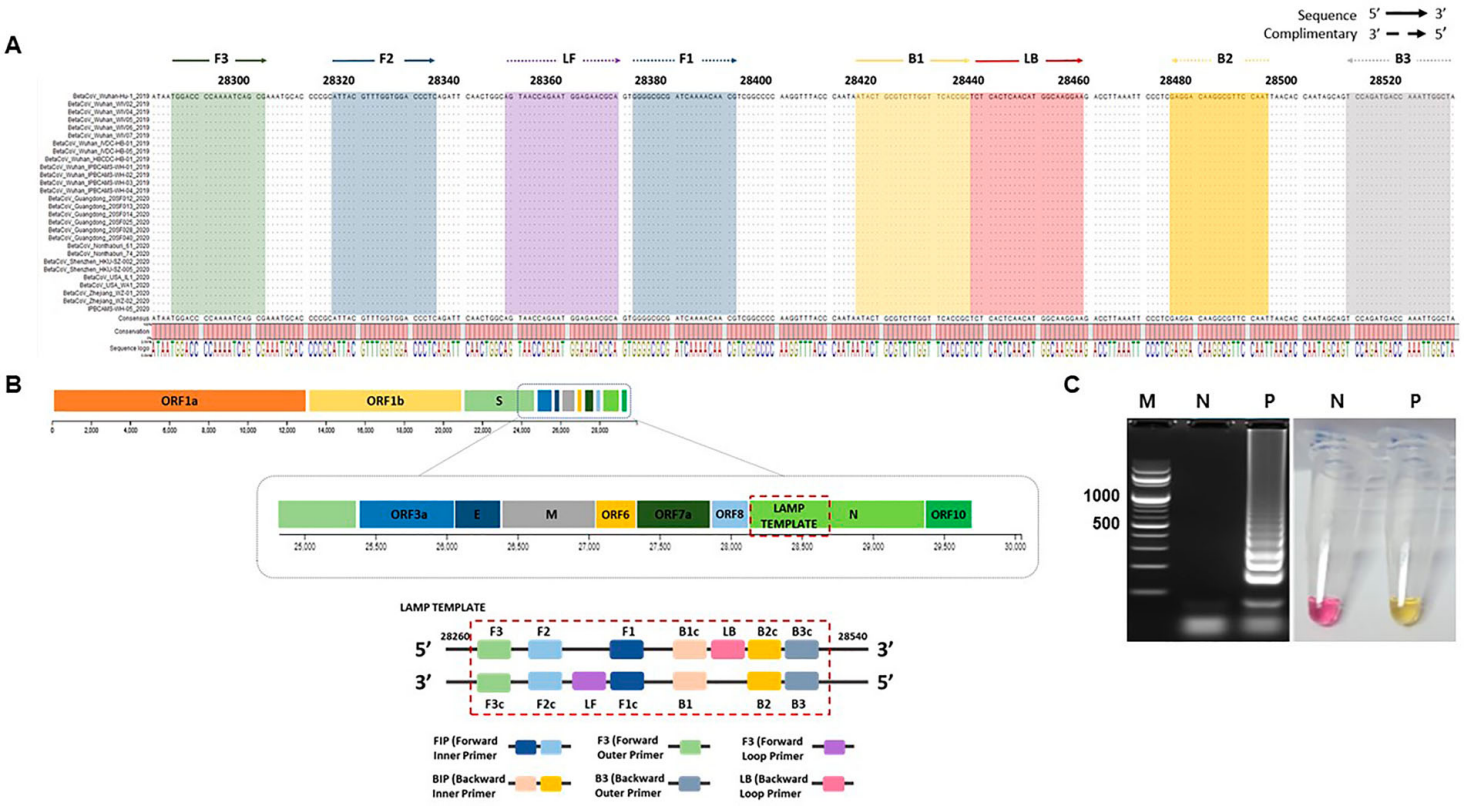
SARS-CoV-2 nucleocapsid (N) gene sequences and RT-LAMP primer designing. A. The sequences were downloaded from GISAID, the alignment of available sequences was done to design the primer sets which targets the N gene. LAMP primers F3, F2, F1, B1, B2, B3 and loop primers LF and LB locations were highlighted as shown. B. LAMP primer sets designed from the target sequences from position 28,285 to 28,529 have a Forward Inner Primer (FIP) and Backward Inner Primer (BIP), both containing sequence complementary (F1c, B1c) to F2 and B2, respectively. C. RT-LAMP optimized using the final selected primer set. The description and sources for each sequence have been indicated in the acknowledgment section. The positive RT-LAMP amplification was optimized using the Primer set enlisted in Table 1. Lane M: 100 bp DNA ladder; Lane N: negative control, lane P: results from the RT-LAMP amplification.

Twenty-eight sequences of SARS-CoV-2 deposited in GISAID were aligned. We designed the oligonucleotide primer sets to target the conserved region of the N-gene sequence which exhibited zero mismatches with the highly conserved region, specifically from position 28,285 to 28,529 (Figure 1(A and B)). We performed the RT-LAMP assay using *in vitro* transcribed 2 μL of 1 ng/mL RNA (1.2 × 10^7^ number of copies) of the SARS-CoV-2 N gene. A successful RT-LAMP reaction results in a colorimetric reaction which includes a change in the colour of phenol red pH indicator from pink to yellow (Figure 1(C)). Moreover, successful amplifications were indicated by a typical ladder-like pattern when electrophoresed in 2% agarose gel (Figure 1(C)).

### Optimization of the RT-LAMP using viral RNA

**Figure 2.**
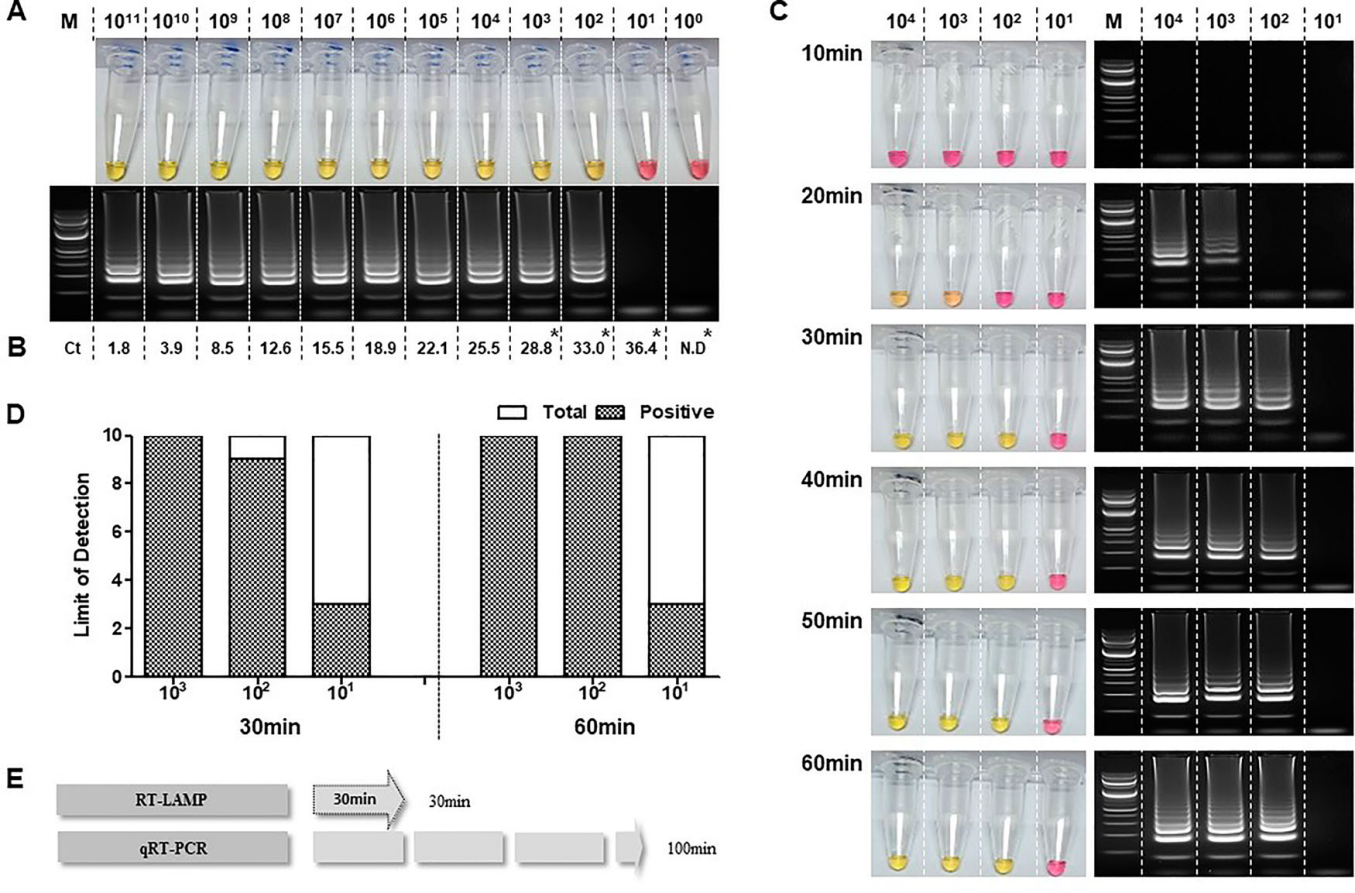
Verifying the sensitivity of the RT-LAMP for viral RNA. A. Sensitivity of the RT-LAMP using RNA ranging from 1 × 10^11^ to 1 × 10^0^ copies as confirmed by the naked eye and 2% agarose gel electrophoresis. B. qRT-PCR positive amplification determined through its cycle threshold value in each RNA-dilution point. C. Determination of optimum reaction time of RT-LAMP for positive amplification that was assessed using the determined dilution limit of SARS-CoV-2 synthesized RNA. Observation of colour change from pink to yellow indicates positive nucleic acid amplification. The left panel shows the RT-LAMP reaction along with the electrophoresed RT-LAMP products for confirmation. (M, 100 bp ladder size marker and serially diluted viral RNA of 10–10,000 concentration of RNA copies). D. Limit of detection in ten repetitions using diluted RNAs (10^3^, 10^2^, and 10^1^). E. Comparative evaluation of time efficiency of RT-LAMP versus qRT-PCR. Lane M: 1000 bp DNA ladder; N.C: negative control, *: Limit of detection of qRT-PCR was evaluated using 10 repeats of 10^3^, 10^2^, 10^1^, and 100 diluted RNA and the results are shown in Supplementary Table 4.

To optimize the RT-LAMP, and determine its limit of detection, a ten-fold serial dilution of the RNA transcript (1 × 10^0^ to 1 × 10^11^ per reaction) was used as the template in both the RT-LAMP (65°C, 60 min) (Figure 2(A)) and qRT-PCR (Figure 2(B)) assays. The standard curve for the qRT-PCR, and associated linear regression, was created using GraphPad Prism (Supplementary Figure 1). For this serial dilution, the RT-LAMP assay was able to detect down to 10^2^ copies per reaction. Ten repetitions of the RT-LAMP assay containing 10^3^, 10^2^, and 10^1^ RNA copies per reaction resulted in 100%, 90%, and 30% detection rate, respectively, while the qRT-PCR detected 100% in 10^3^, 10^2^, and 10^1^ RNA copies and 0% in 10^0^ RNA copies per reaction (Figure 2(A and B)). Nonetheless, compared to RT-LAMP, qRT-PCR method consistently detected up to ten-fold lower (10^1^) RNA copies across ten reactions (qRT-PCR mean Ct=37.43) (Supplementary Table 4).

While discerning the optimal reaction time for the RT-LAMP assay, a clear colorimetric change was evident at 30 min. The clarity of the colorimetric change at 20 min was hard to discern by eye; however, a ladder-like pattern was exhibited through agarose gel electrophoresis (Figure 2(C)). Further assessment between 30 and 60 min incubation times showed 100% positive detection with 10^3^ copies of RNA per reaction to be observed at both time points (across ten repetitions). For 10^2^ copies per reaction, the positive detection rate was 90% for 30 min and 100% for 60 min (across ten repetitions). Moreover, during the ten repetitions, the RT-LAMP was observed to possess an equal detection rate of 30% for detecting 10^1^ RNA copies at both the time points (Figure 2(D)).

In terms of overall time efficiency, excluding the RNA extraction, qRT-PCR takes 100 min to complete and obtain results (Figure 2(E)). The RT-LAMP assay developed in the present study has shown a capability of sensitive detection within 30 min similar to that of 60 min incubation. Hence, for the succeeding evaluation, the RT-LAMP assay was set in 30 min incubation time point.

### Sensitivity evaluation of the RT-LAMP assay in detecting intact viral RNA

**Figure 3.**
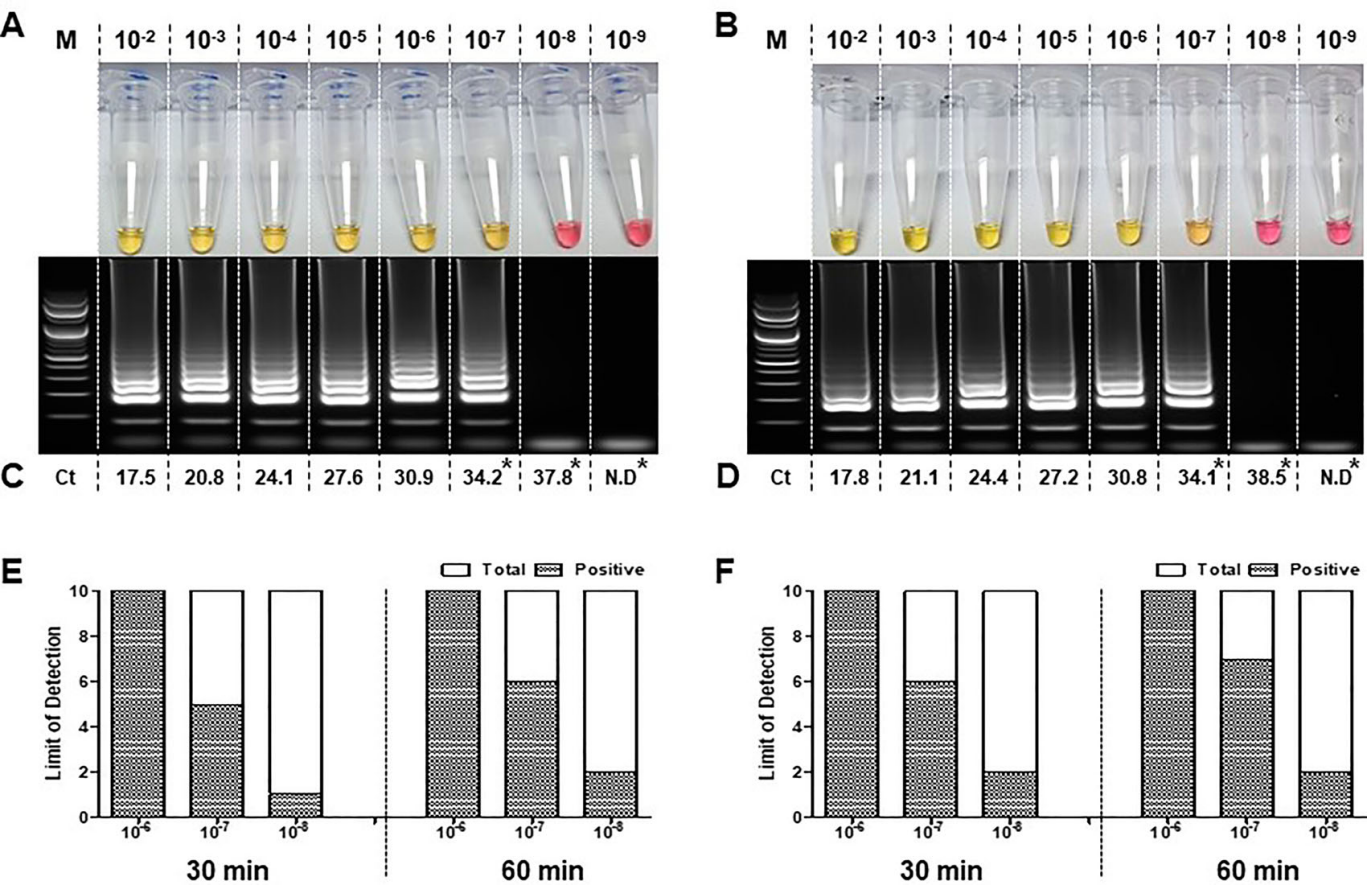
Sensitivity evaluation of the RT-LAMP using intact viral RNA of SARS-CoV-2. RT-LAMP was performed using RNA extracts from cell propagated viruses isolated from two patients diagnosed with COVID-19. Intact viral RNA extracts were ten-fold serially diluted (10^−2^ to 10^−9^) and processed for detection of SARS-CoV-2. RT-LAMP positive reaction results for each virus isolate 1 (A) and isolate 2 (B) were further confirmed through qRT-PCR (C and D). Limit of detection was assessed using 10^−6^, 10^–7^ and 10^–8^ dilutions in ten repetitions, carried out at 65°C for 30 and 60 min incubation (E and D). Change of colour from phenol red to yellow indicates a positive reaction. RT-LAMP products were electrophoresed at 2 % agarose gel for both RNAs. The ladder-like pattern indicates positive nucleic acid amplification. Cycle threshold (Ct) values were also indicated in each figure as a result of the qRT-PCR.; Lane M: 100 bp DNA ladder; N.D: No detection. *: Limit of detection of qRT-PCR was evaluated using ten repeats of 10^−7^, 10^–8^ and 10^–9^ diluted RNA and the results are shown in Supplementary Table 4.

The sensitivity of the RT-LAMP assay was tested to detect the intact viral RNA that was extracted from the cell culture supernatants of two isolates from COVID-19 patients. The sensitivity was observed to be ten-fold lower than that of the qRT-PCR, which was similar to that of the RT-LAMP assay performed using the synthesized RNA. Both assays were able to detect SARS-CoV-2 in a reaction in which intact viral templates were used (Figure 3(A and B)). Interestingly, SARS-CoV-2 positive samples were confirmed in both assays. Both the qRT-PCR and RT-LAMP reactions were able to detect intact viral RNA concentration in the range of 10^−2^ to 10^−7^ dilutions. Significantly, qRT-PCR exhibited ten-fold (10^−8^) higher detection rate for the two intact viral RNA samples. The mean Ct values observed (across three repetitions) are indicated in Figure 3(C and D).

The detection limit of RT-LAMP was determined using ten repetitions of serially diluted templates (10^−6^, 10^−7^ and 10^−8^) for 30 and 60 min incubation (Figure 3 (E and F)). 100% detection was observed in all ten replicates of RNA diluted to 10^−6^ in both the patient isolates regardless of incubation period. However, the RNA samples of 10^−7^ concentrations were observed with lower detection rate of 50-60% in 30 min and 60-70% in 60 min incubation. Moreover, ten replicates of similar serially diluted templates (10^−7^, 10^−8^ and 10^−9^) were subjected to qRT-PCR which demonstrated a ten-fold higher detection rate in agreement with the RT-LAMP results using the synthesized RNA as template (Supplementary Table 4).

### Specificity of the RT-LAMP for viral RNA

**Figure 4.**
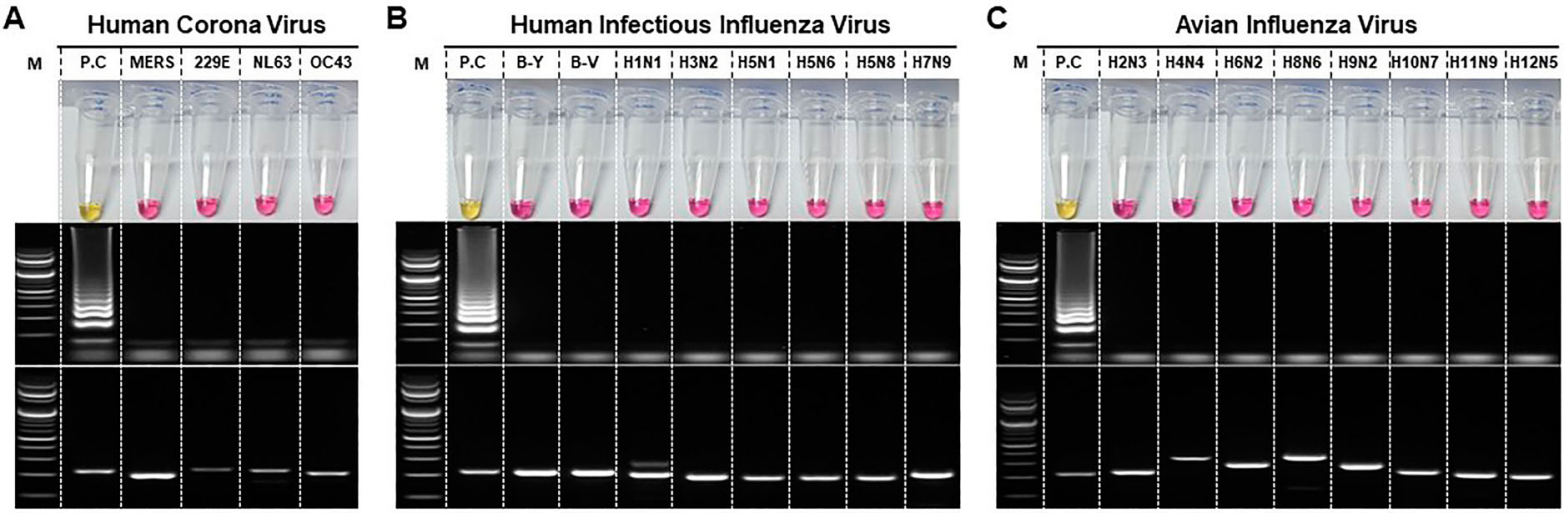
Determination of specificity of the RT-LAMP for SARS-CoV-2. RT-LAMP was performed against panels of A. related coronaviruses, such as human coronaviruses (OC43, NL63, and 229E), and MERS-CoV, B. human infectious influenza viruses, including highly pathogenic avian influenza viruses, and C. avian influenza viruses (low pathogenic). Each set of the panel includes Upper: RT-LAMP, Middle: RT-LAMP products electrophoresed and Lower: One-step RT-PCR Viral RNA confirmation of the samples used in the experiment. Specific primers used for confirmation of amplification of each viral RNA used from various respiratory disease-causing viruses have been indicated in Supplementary Table 2. MERS: Middle East respiratory syndrome coronavirus; B-Y: B/Phuket/3073/2013 (Yamagata lineage); B-V: B/Brisbane/60/2008 (Victoria lineage). Lane M: 1000 bp DNA ladder; PC: SARS-CoV-2 viral RNA (1ng/reaction); Lane N.C: negative control

We have examined the specificities of SARS-CoV-2 RT-LAMP assay against coronaviruses, such as human coronaviruses (OC43, NL63, and 229E), MERS-CoV, influenza viruses, and other respiratory disease-causing viruses. No amplification was observed in RT-LAMP assay for any of the viral RNA panel of related coronaviruses (Figure 4(A). upper panel). This was further confirmed after the gel electrophoresis (Figure 4(A), middle panel). The presence of viral RNA was confirmed for all samples through RT-PCR and gel electrophoresis as shown in the lower panel of Figure 4(A).

Since the signs and symptoms of SARS-CoV-2 are similar to those of influenza virus infection, a panel of highly pathogenic avian influenza viruses and human infectious influenza viruses was tested (Figure 4(B)). No cross-reactivity was observed in RT-LAMP, either through colorimetric detection or agarose gel electrophoresis. Similar results were obtained when the developed RT-LAMP assay was tested using a panel of avian influenza viruses (Figure 4(C)). These results suggest that the developed assay has a high specificity for SARS-CoV-2 against all viruses tested which manifest and/or cause respiratory clinical signs and symptoms.

### Evaluation of the RT-LAMP assay using clinical specimens

A total of 154 clinical samples were evaluated which comprised of 14 positive nasal swab specimens collected from previously diagnosed patients with COVID-19 in National Medical Center, Republic of Korea, and 85 nasal swab specimens collected during the outbreak, and previously confirmed in the hospital for other respiratory disease-causing viruses. In addition, 55 samples confirmed for other respiratory disease-causing viruses and collected prior to the outbreak were also evaluated. In the collected specimens, 16 out of 154 samples were observed to react positively in the RT-LAMP assay (Table 2). Out of 16 samples, 14 were from respiratory samples of COVID-19 patients. Out of 140 negative samples used for this evaluation, two (1.33%) samples were observed to show a positive reaction (Table 2). Using qRT-PCR, we confirmed that both specimens exhibited a false-positive reaction in RT-LAMP (data not shown). We also tested 14 respiratory samples through qRT-PCR. Results revealed that all collected specimens showed positive amplification with Ct value ranging from 21.11 to 32.76. The results of the developed RT-LAMP assay showed a calculated sensitivity of 100% and a specificity of 98.70% which suggests that the primers used in this assay can be used for a sensitive and specific early detection method to identify SARS-CoV-2 cases.

**Table 2.** Specificity of the SARS-CoV-2 RT-LAMP assay using RNA extracted from a clinical specimen.

Virus^a^	Sample type	No. of samples	RT-LAMP	qRT-PCR^b^
Coronavirus				
SARS-CoV-2	Clinical	14	14	21.11–32.76
229E	Clinical	9	–	17.17–38.52
NL63	Clinical	8	1^d^	17.18–40.91
OC43	Clinical	10	–	21.55–37.65
Influenza virus				
Type B	Clinical	30 (29)^c^	–	17.10–34.99
H1N1	Clinical	11	–	15.84–41.15
H3N2	Clinical	30 (26)	–	9.16–34.89
Other respiratory virus				
MPV	Clinical	9	–	19.10–39.49
RSV A	Clinical	1	–	25.11
RSV B	Clinical	15	1^d^	16.77–28.47
PIV	Clinical	3	–	21.73–39.75
AdV	Clinical	3	–	29.08–40.21
HRV	Clinical	11	–	25.61–39.49
Total		154 (55)	16	9.16–41.15

^a^Viruses in abbreviations include SARS-CoV-2: Severe Acute Respiratory Syndrome-Coronavirus-2; 229E: human coronavirus 229E; NL63: human coronavirus NL63; OC43: human coronavirus OC43; Type B: B/Phuket/3073/2013 H1N1pdm: A/California/04/2009; H3N2: A/Perth/16/2009; MPV: human metapneumovirus; RSVA: respiratory syncytial virus A; RSVB: respiratory syncytial virus B; PIV: parainfluenza virus; AdV: Adenovirus; HRV: human rhinovirus.

^b^The cycle threshold values for all analysed viruses when processed in qRT-PCR using their respective primers indicated in the table.

^c^The numbers in parentheses indicate clinical samples collected before SARS-CoV-2 outbreak.

^d^False positive reaction.

## Discussion

In the RT-LAMP method, primers that were designed to target the nucleocapsid (N) protein gene were utilized. In general, the gene encoding N protein was found to have the most abundant expression of subgenomic mRNA (sgmRNA) during infection [35–37]. The coronavirus’ N protein lacks the glycosylation site and possesses distinctly unaltered immunological characteristics [38]. Hence, this protein has been selected as the main target for nucleic acid amplification. Apart from being the most abundant throughout the infection, the gene encoding N protein is highly conserved among all coronavirus structural proteins. Based upon their reported pair-wise patristic distances (PPD) value differences, the emergence of SARS-CoV-2 (PPD: 2.6) was completely independent of SARS-CoV (PPD: 0.16) that emerged in 2002 [24]. Hence, this explains how specific MERS-CoV or SARS-CoV primers limitedly detect this virus and supports why the primers utilized in the RT-LAMP assay are highly specific to SARS-CoV-2 N gene.

The limit of detection revealed that the developed assay is highly capable of detection and nearly as sensitive as qRT-PCR. We have demonstrated that the RT-LAMP assay is specific for SARS-CoV-2 and it possesses rare cross-reactivity with other viruses that manifest similar respiratory disease. Moreover, this assay can discriminate currently co-circulating common human coronaviruses and MERS-CoV. The data presented supports that the RT-LAMP is as specific as qRT-PCR in detecting SARS-CoV-2. In *in silico* analysis, the cross-reactivity between SARS-CoV-2 and SARS-CoV and/or Bat SARS-like CoV would be low based upon the sequence length of the RT-LAMP primer binding regions (10.4-12.3%) (Supplementary Figure 2). In addition, the RT-LAMP assay using the synthesized RNA of SARS-CoV N gene showed positive amplification with 10^7^ RNA copies per reaction (across three repetitions). This result shows 10,000-fold lower cross reactivity compared to that of SARS-CoV-2 N gene (10^2^ RNA copies) (Supplementary Figure 3). Furthermore, using Cohens Kappa, the level of agreement between qRT-PCR and RT-LAMP was revealed to be “almost perfect agreement” (Kappa value = 0.8261) [39]. This supports the high sensitivity and specificity results obtained during the clinical evaluation using both assays.

Generally, RT-LAMP methodology is regarded as new generation diagnostics [40]. However, along with its advantages, this tool may also have some technical shortcomings, such as false-positive single read-out and sensitivity to aerosol contaminants during assay manipulations [31]. The inclusion of RNA extraction step also limits this assay to be genuinely used for bedside testing in patients. Other LAMP methodologies were able to develop assay without any need to conduct RNA extraction, while some utilized a one-step syringe filter system [41,42]. Even though the optimized assay has false positive reactions, we have envisioned that this developed RT-LAMP assay can be utilized as a primary screening method in the rapid detection of SARS-CoV-2. Hence, all positive reactions may be subjected for a subsequent confirmatory diagnosis through qRT-PCR. It would be of best interest if this developed assay be optimized using real-time amplification in order to hasten time reaction and minimize false positive reactions. This RT-LAMP would be an ideal point-of-care-test for COVID-19, if all these limitations are met in future studies.

In summary, we have developed an RT-LAMP assay that can be of use in clinical laboratories in support of the preliminary detection of SARS-CoV-2 in suspected patients especially during the testing of an abundant number of samples. With high sensitivity and specificity, this assay has a simple methodology, low cost, and is time efficient. Further, a single-tube isothermal colorimetric method does not require any expensive equipment. With the declaration of public health of international concerns against SARS-CoV-2, our method would significantly contribute to the public health responses and disease control of countries in providing preliminary counter detection measures, especially to those with limited laboratory capacities.

## Supplementary Material

Supplemental Material
