# Supplementary material for: Development of a reverse transcription-loop-mediated isothermal amplification as a rapid early-detection method for novel SARS-CoV-2
Source: Emerg Microbes Infect. 2020 May 18;9(1):998–1007. doi: 10.1080/22221751.2020.1756698 (PMC7301696; doi:10.1080/22221751.2020.1756698)
Supplement: Supplemental Material [file TEMI_A_1756698_SM0568.docx]

**Supplementary Table 1**. Acknowledgment list of the contributing authors and their respective laboratories for their shared sequences and meta-data of SARS-CoV2 in GISAID on which the primer design of this research was based.

| **Accession ID** | **Virus name** | **Location** | **Collection date** | **Originating lab** | **Submitting lab** | **Authors** |
| --- | --- | --- | --- | --- | --- | --- |
| [EPI_ISL_404227](https://platform.gisaid.org/epi3/start/CoV2020) | BetaCoV/Zhejiang/WZ-01/2020 | Zhejiang, China | 2020-01-16 | Zhejiang Provincial Center for Disease Control and Prevention | Department of Microbiology, Zhejiang Provincial Center for Disease Control and Prevention | Yin Chen, Yanjun Zhang, Haiyan Mao, Junhang Pan, Xiuyu Lou, Yiyu Lu, Juying Yan, Hanping Zhu, Jian Gao, Yan Feng, Yi Sun, Hao Yan, Zhen Li, Yisheng Sun, Liming Gong, Qiong Ge, Wen Shi, Xinying Wang, Wenwu Yao, Zhangnv Yang, Fang Xu, Chen Chen, Enfu Chen, Zhen Wang, Zhiping Chen, Jianmin Jiang, Chonggao Hu |
| [EPI_ISL_404228](https://platform.gisaid.org/epi3/start/CoV2020) | BetaCoV/Zhejiang/WZ-02/2020 | Zhejiang, China | 2020-01-17 | Zhejiang Provincial Center for Disease Control and Prevention | Department of Microbiology, Zhejiang Provincial Center for Disease Control and Prevention | Yanjun Zhang, Yin Chen, Haiyan Mao, Junhang Pan, Xiuyu Lou, Yiyu Lu, Juying Yan, Hanping Zhu, Jian Gao, Yan Feng, Yi Sun, Hao Yan, Zhen Li, Yisheng Sun, Liming Gong, Qiong Ge, Wen Shi, Xinying Wang, Wenwu Yao, Zhangnv Yang, Fang Xu, Chen Chen, Enfu Chen, Zhen Wang, Zhiping Chen, Jianmin Jiang, Chonggao Hu |
| [EPI_ISL_402132](https://platform.gisaid.org/epi3/start/CoV2020) | BetaCoV/Wuhan/HBCDC-HB-01/2019 | China/Hubei Province | 2019-12-30 | Wuhan Jinyintan Hospital | Hubei Provincial Center for Disease Control and Prevention | Bin Fang, Xiang Li, Xiao Yu, Linlin Liu, Bo Yang, Faxian Zhan, Guojun Ye, Xixiang Huo, Junqiang Xu, Bo Yu, Kun Cai, Jing Li, Yongzhong Jiang. |
| [EPI_ISL_402127](https://platform.gisaid.org/epi3/start/CoV2020) | BetaCoV/Wuhan/WIV02/2019 | China / Hubei Province / Wuhan City | 2019-12-30 | Wuhan Jinyintan Hospital | Wuhan Institute of Virology, Chinese Academy of Sciences | Peng Zhou, Xing-Lou Yang, Ding-Yu Zhang, Lei Zhang, Yan Zhu, Hao-Rui Si, Zhengli Shi |
| [EPI_ISL_402128](https://platform.gisaid.org/epi3/start/CoV2020) | BetaCoV/Wuhan/WIV05/2019 | China / Hubei Province / Wuhan City | 2019-12-30 | Wuhan Jinyintan Hospital | Wuhan Institute of Virology, Chinese Academy of Sciences | Peng Zhou, Xing-Lou Yang, Ding-Yu Zhang, Lei Zhang, Yan Zhu, Hao-Rui Si, Zhengli Shi |
| [EPI_ISL_402129](https://platform.gisaid.org/epi3/start/CoV2020) | BetaCoV/Wuhan/WIV06/2019 | China / Hubei Province / Wuhan City | 2019-12-30 | Wuhan Jinyintan Hospital | Wuhan Institute of Virology, Chinese Academy of Sciences | Peng Zhou, Xing-Lou Yang, Ding-Yu Zhang, Lei Zhang, Yan Zhu, Hao-Rui Si, Zhengli Shi |
| [EPI_ISL_402130](https://platform.gisaid.org/epi3/start/CoV2020) | BetaCoV/Wuhan/WIV07/2019 | China / Hubei Province / Wuhan City | 2019-12-30 | Wuhan Jinyintan Hospital | Wuhan Institute of Virology, Chinese Academy of Sciences | Peng Zhou, Xing-Lou Yang, Ding-Yu Zhang, Lei Zhang, Yan Zhu, Hao-Rui Si, Zhengli Shi |
| [EPI_ISL_403963](https://platform.gisaid.org/epi3/start/CoV2020) | BetaCoV/Nonthaburi/74/2020 | Thailand/ Nonthaburi Province | 2020-01-13 | Bamrasnaradura Hospital | 1. Department of Medical Sciences, Ministry of Public Health, Thailand 2. Thai Red Cross Emerging Infectious Diseases - Health Science Centre 3. Department of Disease Control, Ministry of Public Health, Thailand | Pilailuk,Okada; Siripaporn,Phuygun; Thanutsapa,Thanadachakul; Supaporn,Wacharapluesadee; Sittiporn,Parnmen; Warawan,Wongboot; Sunthareeya,Waicharoen; Rome,Buathong; Malinee,Chittaganpitch; Nanthawan,Mekha |
| [EPI_ISL_403962](https://platform.gisaid.org/epi3/start/CoV2020) | BetaCoV/Nonthaburi/61/2020 | Thailand/ Nonthaburi Province | 2020-01-08 | Bamrasnaradura Hospital | 1. Department of Medical Sciences, Ministry of Public Health, Thailand 2. Thai Red Cross Emerging Infectious Diseases - Health Science Centre 3. Department of Disease Control, Ministry of Public Health, Thailand | Pilailuk, Okada; Siripaporn, Phuygun; Thanutsapa, Thanadachakul; Supaporn, Wacharapluesadee; Sittiporn, Parnmen; Warawan, Wongboot; Sunthareeya,Waicharoen; Rome,Buathong; Malinee,Chittaganpitch; Nanthawan,Mekha |
| [EPI_ISL_402119](https://platform.gisaid.org/epi3/start/CoV2020) | BetaCoV/Wuhan/IVDC-HB-01/2019 | China / Hubei Province / Wuhan City | 2019-12-30 | National Institute for Viral Disease Control and Prevention, China CDC | National Institute for Viral Disease Control and Prevention, China CDC | Wenjie Tan, Xuejun Ma, Xiang Zhao, Wenling Wang, Yongzhong Jiang, Roujian Lu, Ji Wang, Peihua Niu,, Weimin Zhou,, Faxian Zhan, Weifeng Shi, Baoying Huang, Jun Liu, Li Zhao, Yao Meng, Fei Ye, Na Zhu, Xiaozhou He, Peipei Liu, Yang Li, Jing Chen, Wenbo Xu, George F. Gao, Guizhen Wu |
| [EPI_ISL_402121](https://platform.gisaid.org/epi3/start/CoV2020) | BetaCoV/Wuhan/IVDC-HB-05/2019 | China / Hubei Province / Wuhan City | 2019-12-30 | National Institute for Viral Disease Control and Prevention, China CDC | National Institute for Viral Disease Control and Prevention, China CDC | Wenjie Tan, Xuejun Ma, Xiang Zhao, Wenling Wang, Yongzhong Jiang, Roujian Lu, Ji Wang, Peihua Niu,, Weimin Zhou,, Faxian Zhan, Weifeng Shi, Baoying Huang, Jun Liu, Li Zhao, Yao Meng, Fei Ye, Na Zhu, Xiaozhou He, Peipei Liu, Yang Li, Jing Chen, Wenbo Xu, George F. Gao, Guizhen Wu |
| [EPI_ISL_402124](https://platform.gisaid.org/epi3/start/CoV2020) | BetaCoV/Wuhan/WIV04/2019 | China / Hubei Province / Wuhan City | 2019-12-30 | Wuhan Jinyintan Hospital | Wuhan Institute of Virology, Chinese Academy of Sciences | Peng Zhou, Xing-Lou Yang, Ding-Yu Zhang, Lei Zhang, Yan Zhu, Hao-Rui Si, Zhengli Shi |
| [EPI_ISL_402123](https://platform.gisaid.org/epi3/start/CoV2020) | BetaCoV/Wuhan/IPBCAMS-WH-01/2019 | China / Hubei Province / Wuhan City | 2019-12-24 | Institute of Pathogen Biology, Chinese Academy of Medical Sciences & Peking Union Medical College | Institute of Pathogen Biology, Chinese Academy of Medical Sciences & Peking Union Medical College | Lili Ren, Jianwei Wang, Qi Jin, Zichun Xiang, Zhiqiang Wu, Chao Wu, Yiwei Liu |
| [EPI_ISL_402125](https://platform.gisaid.org/epi3/start/CoV2020) | BetaCoV/Wuhan-Hu-1/2019 | China | 2019-12 | unknown | National Institute for Communicable Disease Control and Prevention (ICDC) Chinese Center for Disease Control and Prevention (China CDC) | Zhang,Y.-Z., Wu,F., Chen,Y.-M., Pei,Y.-Y., Xu,L., Wang,W., Zhao,S., Yu,B., Hu,Y., Tao,Z.-W., Song,Z.-G., Tian,J.-H., Zhang,Y.-L., Liu,Y., Zheng,J.-J., Dai,F.-H., Wang,Q.-M., She,J.-L. and Zhu,T.-Y. |
| [EPI_ISL_403931](https://platform.gisaid.org/epi3/start/CoV2020) | BetaCoV/Wuhan/IPBCAMS-WH-02/2019 | China / Hubei Province / Wuhan City | 2019-12-30 | Institute of Pathogen Biology, Chinese Academy of Medical Sciences & Peking Union Medical College | Institute of Pathogen Biology, Chinese Academy of Medical Sciences & Peking Union Medical College | Lili Ren, Jianwei Wang, Qi Jin, Zichun Xiang, Zhiqiang Wu, Chao Wu, Yiwei Liu |
| [EPI_ISL_403928](https://platform.gisaid.org/epi3/start/CoV2020) | BetaCoV/Wuhan/IPBCAMS-WH-05/2020 | China / Hubei Province / Wuhan City | 2020-01-01 | Institute of Pathogen Biology, Chinese Academy of Medical Sciences & Peking Union Medical College | Institute of Pathogen Biology, Chinese Academy of Medical Sciences & Peking Union Medical College | Lili Ren, Jianwei Wang, Qi Jin, Zichun Xiang, Zhiqiang Wu, Chao Wu, Yiwei Liu |
| [EPI_ISL_403930](https://platform.gisaid.org/epi3/start/CoV2020) | BetaCoV/Wuhan/IPBCAMS-WH-03/2019 | China / Hubei Province / Wuhan City | 2019-12-30 | Institute of Pathogen Biology, Chinese Academy of Medical Sciences & Peking Union Medical College | Institute of Pathogen Biology, Chinese Academy of Medical Sciences & Peking Union Medical College | Lili Ren, Jianwei Wang, Qi Jin, Zichun Xiang, Zhiqiang Wu, Chao Wu, Yiwei Liu |
| [EPI_ISL_403929](https://platform.gisaid.org/epi3/start/CoV2020) | BetaCoV/Wuhan/IPBCAMS-WH-04/2019 | China / Hubei Province / Wuhan City | 2019-12-30 | Institute of Pathogen Biology, Chinese Academy of Medical Sciences & Peking Union Medical College | Institute of Pathogen Biology, Chinese Academy of Medical Sciences & Peking Union Medical College | Lili Ren, Jianwei Wang, Qi Jin, Zichun Xiang, Zhiqiang Wu, Chao Wu, Yiwei Liu |
| [EPI_ISL_403935](https://platform.gisaid.org/epi3/start/CoV2020) | BetaCoV/Guangdong/20SF025/2020 | Guangdong, China | 2020-01-15 | Guangdong Provincial Center for Diseases Control and Prevention; Guangdong Provincial Public Health | Department of Microbiology, Guangdong Provincial Center for Diseases Control and Prevention | Min Kang, Jie Wu, Jing Lu, Tao Liu, Baisheng Li, Shujiang Mei, Feng Ruan, Lifeng Lin, Changwen Ke, Haojie Zhong, Yingtao Zhang, Lirong Zou, Xuguang Chen, Qi Zhu, Jianpeng Xiao, Jianxiang Geng, Zhe Liu, Jianxiong Hu, Weilin Zeng, Xing Li, Yuhuang Liao, Xiujuan Tang, Songjian Xiao, Ying Wang, Yingchao Song, Xue Zhuang, Lijun Liang, Guanhao He, Huihong Deng, Tie Song, Jianfeng He, Wenjun Ma |
| [EPI_ISL_403934](https://platform.gisaid.org/epi3/start/CoV2020) | BetaCoV/Guangdong/20SF014/2020 | Guangdong, China | 2020-01-15 | Guangdong Provincial Center for Diseases Control and Prevention; Guangdong Provincial Public Health | Department of Microbiology, Guangdong Provincial Center for Diseases Control and Prevention | Min Kang, Jie Wu, Jing Lu, Tao Liu, Baisheng Li, Shujiang Mei, Feng Ruan, Lifeng Lin, Changwen Ke, Haojie Zhong, Yingtao Zhang, Lirong Zou, Xuguang Chen, Qi Zhu, Jianpeng Xiao, Jianxiang Geng, Zhe Liu, Jianxiong Hu, Weilin Zeng, Xing Li, Yuhuang Liao, Xiujuan Tang, Songjian Xiao, Ying Wang, Yingchao Song, Xue Zhuang, Lijun Liang, Guanhao He, Huihong Deng, Tie Song, Jianfeng He, Wenjun Ma |
| [EPI_ISL_403933](https://platform.gisaid.org/epi3/start/CoV2020) | BetaCoV/Guangdong/20SF013/2020 | Guangdong, China | 2020-01-15 | Guangdong Provincial Center for Diseases Control and Prevention; Guangdong Provincial Public Health | Department of Microbiology, Guangdong Provincial Center for Diseases Control and Prevention | Min Kang, Jie Wu, Jing Lu, Tao Liu, Baisheng Li, Shujiang Mei, Feng Ruan, Lifeng Lin, Changwen Ke, Haojie Zhong, Yingtao Zhang, Lirong Zou, Xuguang Chen, Qi Zhu, Jianpeng Xiao, Jianxiang Geng, Zhe Liu, Jianxiong Hu, Weilin Zeng, Xing Li, Yuhuang Liao, Xiujuan Tang, Songjian Xiao, Ying Wang, Yingchao Song, Xue Zhuang, Lijun Liang, Guanhao He, Huihong Deng, Tie Song, Jianfeng He, Wenjun Ma |
| [EPI_ISL_403932](https://platform.gisaid.org/epi3/start/CoV2020) | BetaCoV/Guangdong/20SF012/2020 | Guangdong, China | 2020-01-14 | Guangdong Provincial Center for Diseases Control and Prevention; Guangdong Provincial Public Health | Department of Microbiology, Guangdong Provincial Center for Diseases Control and Prevention | Min Kang, Jie Wu, Jing Lu, Tao Liu, Baisheng Li, Shujiang Mei, Feng Ruan, Lifeng Lin, Changwen Ke, Haojie Zhong, Yingtao Zhang, Lirong Zou, Xuguang Chen, Qi Zhu, Jianpeng Xiao, Jianxiang Geng, Zhe Liu, Jianxiong Hu, Weilin Zeng, Xing Li, Yuhuang Liao, Xiujuan Tang, Songjian Xiao, Ying Wang, Yingchao Song, Xue Zhuang, Lijun Liang, Guanhao He, Huihong Deng, Tie Song, Jianfeng He, Wenjun Ma |
| [EPI_ISL_404895](https://platform.gisaid.org/epi3/start/CoV2020) | BetaCoV/USA/WA1/2020 | USA / Washington / Snohomish County | 2020-01-19 | Providence Regional Medical Center | Division of Viral Diseases, Centers for Disease Control and Prevention | Queen,K., Tao,Y., Li,Y., Paden,C.R., Lu,X., Zhang,J., Gerber,S.I., Lindstrom,S. |
| [EPI_ISL_404253](https://platform.gisaid.org/epi3/start/CoV2020) | BetaCoV/USA/IL1/2020 | USA / Illinois /Chicago | 2020-01-21 | IL Department of Public Health Chicago Laboratory | Pathogen Discovery, Respiratory Viruses Branch, Division of Viral Diseases, Centers for Dieases Control and Prevention | Ying Tao, Krista Queen, Clinton R. Paden, Jing Zhang, Yan Li, Anna Uehara, Xiaoyan Lu, Brian Lynch, Senthil Kumar K. Sakthivel, Brett L. Whitaker, Shifaq Kamili, Lijuan Wang, Janna' R. Murray, Susan I. Gerber, Stephen Lindstrom, Suxiang Tong |
| [EPI_ISL_405839](https://platform.gisaid.org/epi3/start/CoV2020) | BetaCoV/Shenzhen/HKU-SZ-005/2020 | China / Guangdong / Shenzhen | 2020-01 | The University of Hong Kong - Shenzhen Hospital | Li Ka Shing Faculty of Medicine, The University of Hong Kong | Chan,J.F.-W., Yuan,S., Kok,K.H., To,K.K.-W., Chu,H., Yang,J., Xing,F., Liu,J., Yip,C.C.-Y., Poon,R.W.-S., Tsai,H.W., Lo,S.K.-F., Chan,K.H., Poon,V.K.-M., Chan,W.M., Ip,J.D., Cai,J.P., Cheng,V.C.-C., Chen,H., Hui,C.K.-M. and Yuen,K.Y. |

**All submitters of data may be contacted through www.gisaid.org.

**Supplementary Table 2.** Sequence and primers for synthesized SARS-CoV-2 RNA

| **Target:** | **Primer Name** | **Sequence** | **Length (mer)** |
| --- | --- | --- | --- |
| Synthesized SARS-CoV-2  N gene^A^ | T7-SARS-CoV-2 N gene-F^C^ | TAATACGACTCACTATAGGGTATCATGACGTTCGTGTTGTTTTA | 44 |
|  | T7-SARS-CoV-2 N gene-R | GGCCCAGTTCCTAGGTAGTA | 20 |
| Synthesized SARS-CoV  N gene^B^ | T7-SARS-CoV_N-F^C^ | TAATACGACTCACTATAGGGGCATTTAGAGACGTACTTGTTGT | 43 |
|  | T7-SARS-CoV_N-R | GGGCCAGTTCCTAGGTAA | 18 |

^A^ SARS-CoV-2 N gene sequence was based on BetaCoV/Wuhan-Hu-1/2019, (GISAID, Accession ID: EPI_ISL_402125)

^B^ SARS-CoV-2 N gene sequence was based on SARS-CoV HKU-39849, (GenBank, Accession no.: AY278491.2)

^C^ T7-RNA polymerase sequence was underlined in the sequence designed.

|  | **Virus** | **Primer** | **Sequence (5' to 3')** | **Size** |
| --- | --- | --- | --- | --- |
| Corona virus | SARS-CoV-2 | nCoV-F3 | TGGACCCCAAAATCAGCG | 209 |
|  |  | nCoV-B3 | AGCCAATTTGGTCATCTGGA |  |
|  | MERS | MERS F3-2 | GGAATGGAATTAAGCAACTGGC | 190 |
|  |  | MERS B3-2 | CGCGAATTGTGTAACAATAGCT |  |
|  | 229E | multi-229E | TTAAGCAGTATACTTCTGCTTGT | 124 |
|  |  | multi-229E | GGCAAAACCATTATGCCATTGTA |  |
|  | NL63 | NL63 S F | GCAAAACTTACTACAGTTAC | 200 |
|  |  | NL63 S R | GGAGGTACTCAACTTGAACT |  |
|  | OC43 | OC43 380F | TGTGATGGTGGATTGTCGCCG | 203 |
|  |  | OC43 583R | TCCTTTAGGATTACAACCTCTCAC |  |
| Influenza virus | B | B-F3 | CAGGAAGAGTAAAACATACTGAGGA | 213 |
|  |  | B-B3 | GATTCGCAAGGCCCTGTT |  |
|  | H1 | H1-F3 | AGCAAGAAGTTCAAGCCG | 201 |
|  |  | H1-B3 | CGTGAACTGGTGTATCTGAA |  |
|  | H3 | H3-F3 | GGGGTTACTTCAAAATACGAAG | 188 |
|  |  | H3-B3 | GTTGCCAATTTCAGAGTGTT |  |
|  | H5 | H5-F3 | GCTATAGCAGGTTTTATAGAGG | 181 |
|  |  | H5-B3 | GCCTCAAACTGAGTGTTCAT |  |
|  | H7 | H7-F3 | GCGGGTTTCATTGAAAATGG | 201 |
|  |  | H7-B3 | CTACCTCATTGAATTCATTGTCT |  |
|  |  |  |  |  |
| Avian influenza virus | H2 | H2 199F | TTGGGAGATTGCAGCATCGC | 227 |
|  |  | H2 426R | TGTTGTATGCTGTGTCCACC |  |
|  | H4 | H4 746F | TCGTAGAGCCTGGAGACCTA | 311 |
|  |  | H4 1057R | AAAGAGCCCTCTTGATGCCT |  |
|  | H6 | H6 693F | CAGCAAGACCTGCTGTGAAC | 280 |
|  |  | H6 973R | CACATATTTGGGGCATTCTC |  |
|  | H8 | H8 980F | AAAGCCTACGGCTGGCAGTT | 336 |
|  |  | H8 1316R | GAGGACCAGAAGCTCTGCAT |  |
|  | H9 | H9 1262F | AGATTGATGACCAAATACAG | 279 |
|  |  | H9 1541R | ACCCCTTCTATTTTCTGCCT |  |
|  | H10 | H10 256F | CATCTTACAGGGACATGGGA | 229 |
|  |  | H10 485R | AGCCACTTGAGCTCTGCGTA |  |
|  | H11 | H11 503F | ACCAATCAGGAACATACCCT | 215 |
|  |  | H11 718R | CAGCCTGTCCATTAACTTTG |  |
|  | H12 | H12 565F | GTATTCACATGGGCTATTCA | 188 |
|  |  | H12 753R | GTATCTTCACTGTCTGCCCT |  |

**Supplementary Table 3.** List of specific primers used to confirm the identity of various clinical specimen used in the optimization and clinical evaluation of RT-LAMP

** One-step RT-PCR and qRT-PCR were processed under the following conditions: reverse transcription at 55 °C for 30 min, initial denaturation at 95 °C for 5 min, 35 cycles of denaturation at 95 °C for 30s, annealing at 60 °C for 30s, elongation at 72 °C for 30s, and a final elongation step at 72 °C for 5 min.

**Supplementary Table 4.** Confirmation of limit of detection of synthesized and viral intact SARS-CoV-2 RNA processed in qRT-PCR using reference probes shared by NIID-Japan [33].

| Sample tested in qRT-PCR | Viral RNA concentration | No. of replicates with positive amplification | Mean Ct value^*^ | SD^*^ |
| --- | --- | --- | --- | --- |
| Synthesized SARS-CoV-2 RNA | 1x10^3^ | 10/10 | 28.79 | 0.34 |
|  | 1x10^2^ | 10/10 | 32.38 | 0.85 |
|  | 1x10^1^ | 10/10 | 37.43 | 1.26 |
|  | 1x10^0^ | 0/10 | ND | ND |
| Intact Viral RNA from Isolate 1 | 1x10^-7^ | 10/10 | 34.36 | 0.34 |
|  | 1x10^-8^ | 6/10 | 37.15 | 1.33 |
|  | 1x10^-9^ | 0/10 | ND | ND |
| Intact Viral RNA from Isolate 2 | 1x10^-7^ | 10/10 | 34.66 | 0.54 |
|  | 1x10^-8^ | 8/10 | 37.09 | 1.39 |
|  | 1x10^-9^ | 0/10 | ND | ND |

^*^Values were calculated using GraphPad Prism; ND means not detected and no viral amplification observed.


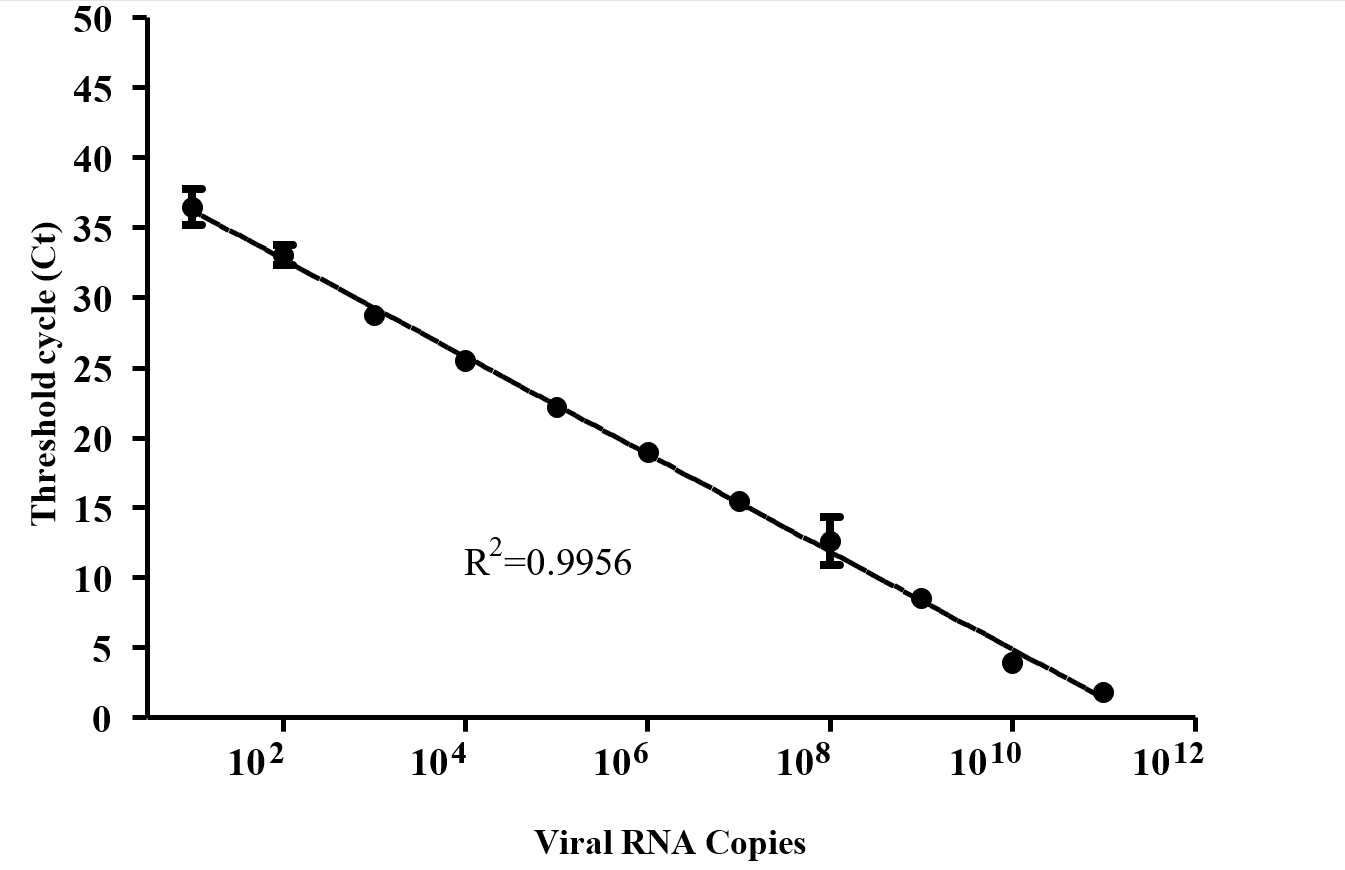


| Dilution | Mean Ct value | Standard deviation |
| --- | --- | --- |
| 10^1^ | 36.43 | 1.27 |
| 10^2^ | 33.00 | 0.72 |
| 10^3^ | 28.77 | 0.15 |
| 10^4^ | 25.50 | 0.00 |
| 10^5^ | 22.13 | 0.15 |
| 10^6^ | 18.93 | 0.15 |
| 10^7^ | 15.47 | 0.21 |
| 10^8^ | 12.60 | 1.73 |
| 10^9^ | 8.50 | 0.26 |
| 10^10^ | 3.93 | 0.51 |
| 10^11^ | 1.80 | 0.26 |

**Supplementary Figure 1.** Tenfold serial dilutions ranging from 1x101 to 1x1011 copies of SARS-CoV-2 copies of RNA. The standard curve graph is made by plotting the mean cycle threshold values (Ct) on the y-axis and the log input amounts on the x-axis. The slope of the standard curve is -3.479, and the correlation coefficient is 0.9956. The standard deviation for each mean Ct values was presented in the figure. Error bars for SD values were added in the points of the linear regression slope.


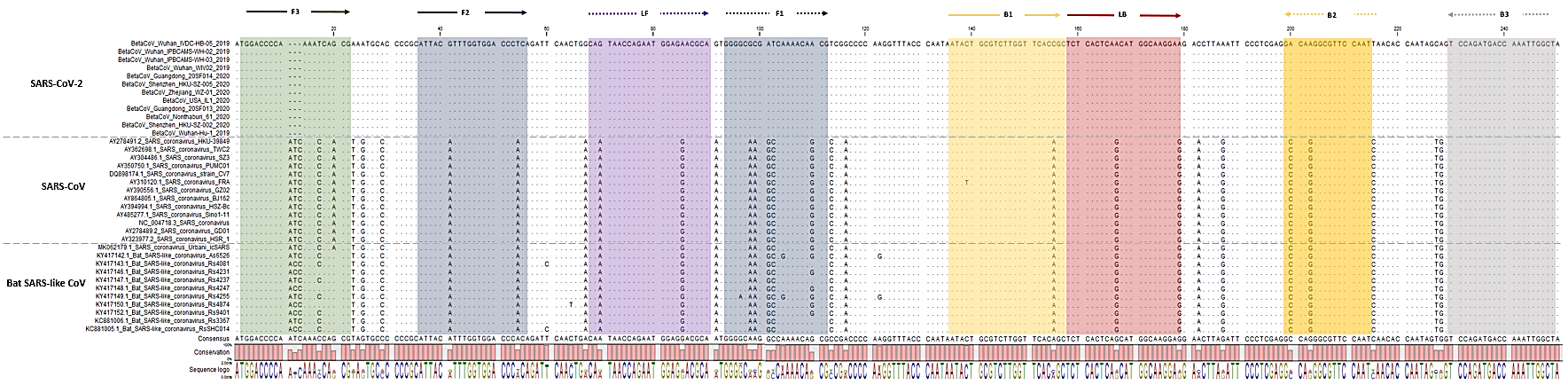

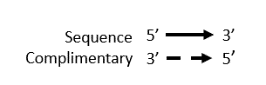


**Supplemental Figure 2: Comparative alignment of SARS-related coronavirus sequences in the specific primer region of the developed RT-LAMP.** Using the sequences downloaded from GISAID and GenBank, the representative sequences for SARS-CoV-2, SARS-CoV and Bat SARS-like CoV were aligned to compare the sequence differences specially in the primer region selected for the developed RT-LAMP. The target regions for each of the designed RT-LAMP primers (F3, F2, F1, B1, B2, B3 and loop primers LF and LB) were highlighted as shown. Matching residues were represented as dots. The authors acknowledge the authors of the sequences used in this supplementary data. SARS-CoV-2 sequences were all downloaded from GISAID as acknowledged previously while SARS-CoV (Accession nos.: AY278491.2; AY362698.1; AY304486.1; AY350750.1; DQ898174.1; AY310120.1; AY390556.1; AY864805.1; AY394994.1; NC_004718.3; AY278489.2; and AY 323977.2) and BAT SARS-Like CoV (Accession nos.: MK062179.1; KY417142.1; KY417143.1; KY417146.1; KY417147.1; KY417148.1; KY417149.1; KY417150.1; KY417152.1; KC881006.1 and KC881005.1) were downloaded from GenBank (<https://www.ncbi.nlm.nih.gov/genbank/>).


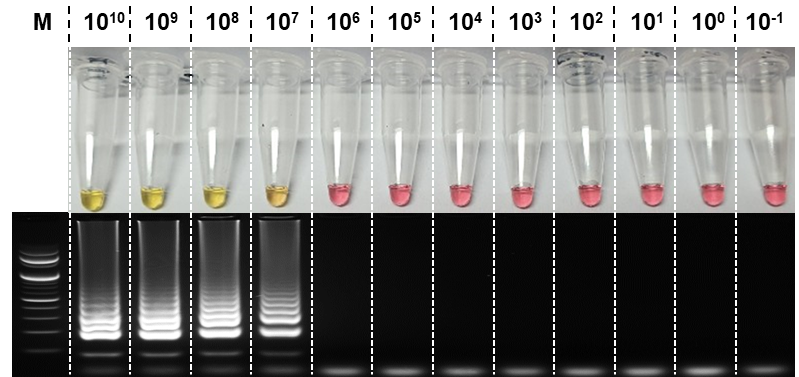


**Supplemental Figure 3: Determination of the RT-LAMP’s cross-reactivity for SARS-CoV.** Ten-fold serial dilution (1010 to 10-1) of SARS-CoV synthesized RNA was tested using the developed RT-LAMP incubated for 30 min at 65ºC. RT-LAMP observation of color change from pink to yellow indicates positive nucleic acid amplification. Top panel shows the RT-LAMP reaction along with the electrophoresed RT-LAMP products (bottom panel) for confirmation. (M, 100 bp ladder size marker and serially diluted viral RNA of 1010 to 10-1 concentration of SARS-CoV RNA copies.
